# Modulation of polymorphonuclear neutrophil functions by astrocytes

**DOI:** 10.1186/1742-2094-7-53

**Published:** 2010-09-09

**Authors:** Luokun Xie, Ethan C Poteet, Wenjun Li, Amanda E Scott, Ran Liu, Yi Wen, Anuja Ghorpade, James W Simpkins, Shao-Hua Yang

**Affiliations:** 1 Department of Pharmacology and Neuroscience, University of North Texas Health Science Center at Fort Worth, Fort Worth, TX. 76107, USA; 2 Institute for Aging and Alzheimer's Disease Research, University of North Texas Health Science Center at Fort Worth, Fort Worth, TX. 76107, USA; 3 Department of Cell Biology and Anatomy, University of North Texas Health Science Center at Fort Worth, Fort Worth, TX. 76107, USA; 4 Center for Autoimmune and Musculoskeletal Disease, the Feinstein Institute for Medical Research, 350 Community Drive, Manhasset, NY 11030, USA

## Abstract

**Background:**

Neuroinflammation is a complex process involving cells from the immune system and the central nerve system (CNS). Polymorphonuclear neutrophils (PMN) are the most abundant class of white blood cells, and typically the first type of leukocyte recruited to sites of inflammation. In the CNS, astrocytes are the most abundant glial cell population and participate in the local innate immune response triggered by a variety of insults. In the present study, we investigated the impacts of astrocytes on PMN function.

**Methods:**

Primary astrocyte cultures were derived from postnatal C57BL/6 mice and primary neutrophils were isolated from 8 to 12 weeks old C57BL/6 mice. PMNs respiratory burst was analyzed by H2DCFDA assay. For phagocytosis assay, neutrophils were incubated with FITC-labeled E. coli and the phagocytosis of E coli was determined by flow cytometer. PMNs degranulation was determined by myeloperoxidase assay. Cytokine expression was determined by real-time PCR. To determine the involvement of different signaling pathway, protein lysates were prepared and western blots were conducted to assess the activation of Akt, Erk1/2, and p38.

**Results:**

Using ex vivo neutrophils and primary astrocyte cultures, our study demonstrated that astrocytes differentially regulate neutrophil functions, depending upon whether the interactions between the two cell types are direct or indirect. Upon direct cell-cell contact, astrocytes attenuate neutrophil apoptosis, respiratory bust, and degranulation, while enhancing neutrophil phagocytic capability and pro-inflammatory cytokine expression. Through indirect interaction with neutrophils, astrocytes attenuate apoptosis and enhance necrosis in neutrophils, augment neutrophil phagocytosis and respiratory burst, and inhibit neutrophil degranulation. In addition, astrocytes could augment Akt, Erk1/2, and p38 activation in neutrophils.

**Conclusions:**

Astrocytes differentially regulate neutrophil functions through direct or indirect interactions between the two cell types. The diversified actions of astrocytes on neutrophils might provide protection against potential microbial infections given compromised blood-brain barrier integrity under certain neuropathological conditions. The complex actions of astrocytes on neutrophils could provide further insight to harness the inflammatory response to promote CNS repair.

## Background

Inflammation is a complex set of interactions among soluble factors and cells that can arise in any tissue in response to infection and tissue damage induced by ischemic, traumatic, or autoimmune injury. The process normally leads to recovery and healing. However, inflammation loses its repairing function and can enhance tissue damage if this process is not properly controlled [1]. Polymorphonuclear neutrophils are the most abundant class of white blood cells, and typically the first type of leukocyte recruited to sites of inflammation. It is well understood that neutrophils play an important role in the battle against invading pathogens by delivery of antimicrobial molecules and reactive oxygen intermediates [2]. Inflammation is also recognized as a major contributor to diverse acute and chronic central nervous system (CNS) disorders. Neuroinflammation is a complex process involving cells from both peripheral immune system and CNS and plays an important role in the pathogenesis of neurological diseases. For chronic neurodegenerative disorders like Alzheimer's disease and Parkinson's disease, neuroinflammation mostly involves activation of the innate immune responses in the brain [3,4]. On the other hand, invasion of peripheral immune cells and interactions between innate and systemic immune cells occurs in acute brain damages as stroke, infection, and traumatic brain injury. Studies have shown the existence of neutrophils in the CNS under these pathological conditions [5-9]. Given the abundance of neutrophils in the CNS during neuroinflammation and the non-specificity of the neutrophil-derived cytotoxic compounds, it is speculated that neutrophils' anti-infectious action in host defense can also mediate tissue damages. From an evolutionary view, the homeostasis of neutrophils must be highly regulated. Indeed, the inflammation response is a complex process and includes production of host-derived anti-inflammatory mediators and apoptosis of neutrophils [10]. In addition, function of the infiltrated neutrophil might be regulated by the local innate immune cells. Consistently, it has been demonstrated that microglia could interact and engulf invading neutrophils, hence providing protection for the CNS [11].

Astrocytes are the most abundant glial cell population in the CNS and play a critical role for the maintenance of CNS homeostasis. Evidence is emerging that astrocytes participate in the local innate immune response triggered by a variety of insults [12]. Astrocytes modulate nitric oxide production by microglia through secretion of serine and glycine [13]. Under inflammatory conditions, intercellular communication in the astroglial network may be regulated by activated microglia. Microglia-derived Prostaglandin D_2 _enhances GFAP production and astrogliosis [14]. Studies have also demonstrated that astrocytes could regulate peripheral immune cells that infiltrated into the CNS under pathological conditions. In response to T cell attack, astrocytes polarize towards the contacting T cells in vivo and in vitro [15], and present antigens to T cells [16]. Astrocytes may suppress antigen-dependent T cell proliferation as well as cytokine production [17,18]. Surprisingly, interactions between astrocytes and neutrophils, the most abundant cell types in the CNS and in the peripheral immune system, respectively, has not been well characterized. In the present study, we focused on the interaction of astrocytes and neutrophils to investigate the impact of astrocytes on different functions of neutrophils using *ex vivo *cultured mouse primary astrocytes and bone marrow-derived neutrophils.

## Methods

### Experimental animals

C57/B6 mice were purchased from Charles River. All animal procedures were approved by the University of North Texas Health Science Center Animal Care and Use Committee.

### Isolation of polymorphonuclear neutrophils

Murine bone marrow neutrophils were isolated using flow cytometry sorting and magnetic isolation methods. Briefly, femurs and tibias were isolated from 8~12-week old male C57BL/6 mice. The hematopoietic cells were obtained by flushing the bone marrow cavity using a 26-gauge needle. Red blood cells were lysed with BD Pharm Lyse™ Lysing Buffer. Then the hematopoietic cells were stained with Allophycocyanin (APC) conjugated anti-mouse Ly-6G (Gr1) monoclonal antibody (eBioscience) and were subjected for FACS sorting using a BD Cytopeia InFlux Cell Sorter. For magnetic isolation, bone marrow cells were first labeled with biotinylated anti-mouse Ly-6G (Gr1) monoclonal antibody (eBioscience) followed by incubation with streptavidin-coated Dynabeads (Invitrogen) according to manufacturer's instruction. Gr1^+ ^cells were collected on a DynaMagTM-Spin concentrator (Invitrogen). All Gr1^+ ^cells were suspended in RPMI-1640 (GIBCO) supplemented with 10% FBS, 100 μg/ml penicillin and streptomycin for further treatment.

### Primary culture of murine astrocytes

Day-2 postnatal C57BL/6 mouse pups were sacrificed according to current college IACUC guidelines. Brains were removed and meninges were peeled off. Cortices were dissected out in a 10 cm Petri dish containing 5 ml of phenol red-free DMEM (GIBCO) supplemented with 100 μg/ml penicillin and streptomycin. Then cortices were centrifuged at 2200 rpm for 5 min and supernatant was aspirated. An equal volume of 0.25% trypsin-EDTA was added to resuspend the pellets and incubated for 20 min with gentle agitation every 5 min. After digestion, tissues were spun down and supernatants were aspirated. Tissues were resuspended in an equal volume of DMEM. Cells were dissociated by pipetting up and down 10 to 12 times using a fire-polished Pasteur pipette. Following the dissociation, suspension was filtered through a 40 μm cell strainer into a 50 ml conical tube and cell number was counted. About 10 × 10^6 ^cells were seeded into 400 ml flasks containing DMEM supplemented with 10% FBS, 100 μg/ml penicillin and streptomycin. Cells were incubated for 10~14 days in a humidified atmosphere of 5% CO2 in air at 37°C until cultures were confluent. Cultures were shaken for 6 hours at 37°C at 250 rpm on an orbital shaker and floating cells were discarded after shaking. Adhesive cells were trypsinized and cell density was adjusted before use.

### Co-culture of neutrophils with astrocytes

Astrocytes (5 × 10^5^/ml) were seeded into flat-bottom tissue culture plates and incubated in the presence or absence of 25 μg/ml poly I:C (Sigma) for 24 hours, followed by 3 washes with PBS. Fresh supplemented DMEM was added immediately before co-culture. Neutrophils (1 × 10^6^/ml) were incubated for 2 hours in the presence or absence of 100 ng/ml lipopolysaccharide (LPS) or 1 μM N-Formyl-L-methionyl-L-leucyl-L-phenylalanine (fMLP) as stimuli. Unless indicated, cells were vigorously washed with PBS to remove stimuli and were resuspended with fresh supplemented RPMI-1640 at a density of 1 × 10^6^/ml. Then neutrophils were seeded into astrocyte-containing tissue culture plates at a ratio of 1:1 (PMNs: astrocytes) and incubated for the indicated period of time. For control, neutrophils were seeded in fresh DMEM without astrocyte. After co-culture, all cells were collected and subjected for further sorting or analysis.

### Immunofluorescent staining

For immunocytochemistry, cultured cells were fixed with methanol for 10 min at 4°C and were incubated in blocking buffer (10% goat serum-TBST) for 30 min. After 3 washes with TBS, sections were incubated with 2 μg/ml primary antibodies diluted in 0.5% BSA-TBS overnight at 4°C. Culture cells were washed 3 times with TBS and incubated with fluorochrome-conjugated secondary antibodies for 1 hour at room temperature. After 3 washes with TBS, culture cells were mounted with ProLong^® ^Gold antifade reagent with DAPI (Molecular Probes) and coverslips.

### Cell-cell adhesion analysis

To detect cellular interaction between neutrophils and primary astrocytes in a floating culture system, astrocytes were labeled with 5 μM 5-(and-6)-carboxyfluorescein diacetate, succinimidyl ester (CFSE, Invitrogen) for 15 min at 37°C. Labeled astrocytes were incubated in the presence or absence of 25 μg/ml poly I:C for 24 hr. Astrocytes were trypsinized with 0.25% trypsin-EDTA and washed with PBS. The cells were resuspended in DMEM at a density of 5 × 10^5^/ml. Sorted Gr1^+ ^neutrophils were treated with or without indicated stimuli for 2 hr before assay. Then neutrophils were washed 3 times with PBS and resuspended in RPMI-1640 at a density of 2.5 × 10^6^/ml. Twenty five μl of astrocyte suspension was mixed with 25 μl of neutrophil suspension (PMN:astrocytes = 5:1) followed by incubation with periodical agitation for indicated periods of time at 37°C. Binding of neutrophils to astrocytes was determined as CFSE^+ ^and Gr1^+ ^cells by flow cytometry analysis.

To determine the binding between neutrophils and primary astrocytes in a static culture system, 5 × 10^5^/ml astrocytes were seeded into a 96-well culture plate (Corning) and incubated in the presence or absence of 25 μg/ml poly I:C for 24 hr. Then the supernatants were aspirated and cells were washed 3 times with PBS. Sorted neutrophils were labeled with 5 μM CFSE and resuspended at a density of 2.5 × 10^6^/ml in RPMI-1640. One hundred μl of neutrophils were added into each well to mix with astrocytes and incubated for 1.5 hr. Non-adherent cells were washed away and CFSE fluorescence was measured using a Tecan Infinite M200 microplate reader.

### Respiratory burst detection

PMNs respiratory burst was tested with 2, 7-dichlorodihydrofluorescein diacetate (H2DCFDA, Invitrogen), a cell-permeable indicator for reactive oxygen species (ROS), following manufacture's instruction. Briefly, resting or stimulated neutrophils were incubated in pre-warmed PBS containing 0.5% BSA and 10 μM H2DCFDA for 15 min at 37°C, followed by washing with PBS. Cells were then resuspended in RPMI-1640 for further incubation. The ROS production was determined by measuring the green fluorescence using a BDTM LSRII flow cytometer.

### Phagocytosis assay

FITC-labeled E. coli were prepared by the method described previously [19]. Heat-killed bacteria (1 × 10^10 ^bacteria/ml) were washed 3 times with 0.85% saline and then resuspended in 0.85% saline. The suspension was adjusted to reach an optical density of 2.0 at a wavelength of 540 nm. One volume of bacterial suspension was diluted with 5 volumes of the hydrogen phosphate/dihydrogen phosphate buffer (pH 9.6) consisting of 0.1 M Na_2_HPO4 and 0.1 M NaH_2_PO4. Fluorescein 5-isothiocyanate [isomer 1] (FITC, Sigma) was prepared as a 0.3 mg/ml solution in the same hydrogen phosphate/dihydrogen phosphate buffer (pH 9.6). Two volumes of the FITC solution were then added to the bacterial suspension. This suspension was incubated at room temperature in darkness for 2 hr followed by being washed three times in veronal buffered saline (pH 8.6) containing 0.1 M sodium diethyl barbiturate, 0.15 mM CaCl2, 1 mM MgSO4, 0.1% gelatin. Bacteria were then resuspended in the same buffer and were aliquoted at 2 × 10^9^/ml and stored at -20°C.

Bacteria were opsonized in mouse serum for 30 min at 37°C. The bacteria were washed three times with PBS and resuspended in RPMI 1640 at a concentration of 2.5 × 10^9^/ml. 10 μl of bacteria were mixed with 5 × 10^5 ^neutrophils (neutrophils: E. coli ratio of 1:50) and incubated on ice for 30 min with periodical agitation to allow attachment of E. coli to neutrophils. Then, cells with bacteria were incubated for 15 min at 37°C. Equal volume of 0.4% trypan blue-PBS was added to quench the extracellular fluorescence before neutrophils were subjected to flow cytometry analysis.

### Degranulation assay

PMNs degranulation was determined using a myeloperoxidase assay kit (Cytostore) according to manufacture's instruction. Supernatants from neutrophil culture were mixed with equal volume of sample buffer containing 5 mg/ml hexadecyltri-methylammonium bromide. Twenty μl of mixture was added into 200 μl of development buffer and absorbance at 450 nm was measured after incubation for 10 min at 37°C.

### Quantitative real-time PCR

PMN RNAs were extracted using Trizol LS Reagent according to manufacture's instruction. 10 μl of RNA solution was mixed with 0.5 μl of random primer (Promega) and 0.5 ul of RNAse inhibitor (Invitrogen). The mixture was then heated for 3 min at 70°C and placed on ice. Following reagents were added to the mixture to get a total reaction volume of 20 μl: 4 μl of 5× reaction buffer (USB), 2 μl of 0.1 M DTT, 2 μl of 10 mM dNTP Mix and 1 μl of M-MuLV reverse transcriptase (USB). The mixture was incubated for 10 min at 25°C, followed by subsequent incubation for 60 min at 42°C and final incubation for 15 min at 70°C. Synthesized cDNAs were stored at -20°C.

Quantitative real-time PCR was carried out using SYBR Green PCR Master Mix (Molecular Probes), 7300 Real-Time PCR System from Applied Biosystems. 1 μl of RT product was mixed with 10 μl of SYBR Green PCR Master Mix, 0.4 μl of ROX reference dye, 8.2 μl of deionized water and specific primers (300 nM) in a total volume of 20 μl. The reactions in 96-well plates were held at 50°C for 2 min and 95°C for 10 min, followed by 40 thermal cycles at 95°C for 15 s and 64°C for 1 min. The quantification of the target genes was analyzed using the included software and normalized against mouse β-actin RNA. Sequences of specific primers are as follows:

CXCL2: Forward 5' gccaagggttgacttcaagaac 3'

Reverse 5' aggtcagttagccttgcctttg 3'

TNF-α: Forward 5' gcctcttctcattcctgcttgt 3'

Reverse 5' caggcttgtcactcgaattttg 3'

IL-1β: Forward 5' ggtgtgtgacgttcccattaga 3'

Reverse 5' tcgttgcttggttctccttgta 3'

IL-6: Forward 5' gacttccatcgagttgccttct 3'

Reverse 5' ttgggagtggtatcctctgtga 3'

### Western Blot

PMNs were isolated with Dynabeads and were co-cultured with astrocytes at a ratio of 3:1 for indicated periods. Neutrophils were then collected in cold PBS containing 2 mM EDTA and 0.5% bovine serum albumin (BSA) followed by isolation on a magnetic concentrator again to get rid of astrocytes. Microscopy showed few astrocytes in the cell suspension after isolation. Neutrophils were lysed and proteins were separated by Tris-Glycine polyacrylamide gel. The proteins were transferred to a nitrocellulose membrane, and the membrane were blocked for 1 hour with 5% non-fat dry milk in PBS and incubated over night at 4°C with primary antibodies. The membranes were repeatedly washed with PBS prior to incubation with secondary horseradish peroxidase-conjugated secondary antibodies. The blots were developed with an enhanced chemiluminescence reagent. The images were acquired and analyzed by a Western blot imaging system (UVP, upland, CA).

### Statistics

All values were expressed as mean ± standard error of mean (SEM). Binding between astrocytes and neutrophils, apoptosis of neutrophils, phagocytosis of neutrophils, ROS production of neutrophils, MPO releasing, and real time PCR data were analyzed by two-way ANOVA. When a significant difference was detected by ANOVA, a post hoc Tukey's test was performed to identify a specific difference between groups. N represents independent cultures or experiments.

## Results

### Astrocytes mediate alteration of neutrophils morphology

**Figure 1:**
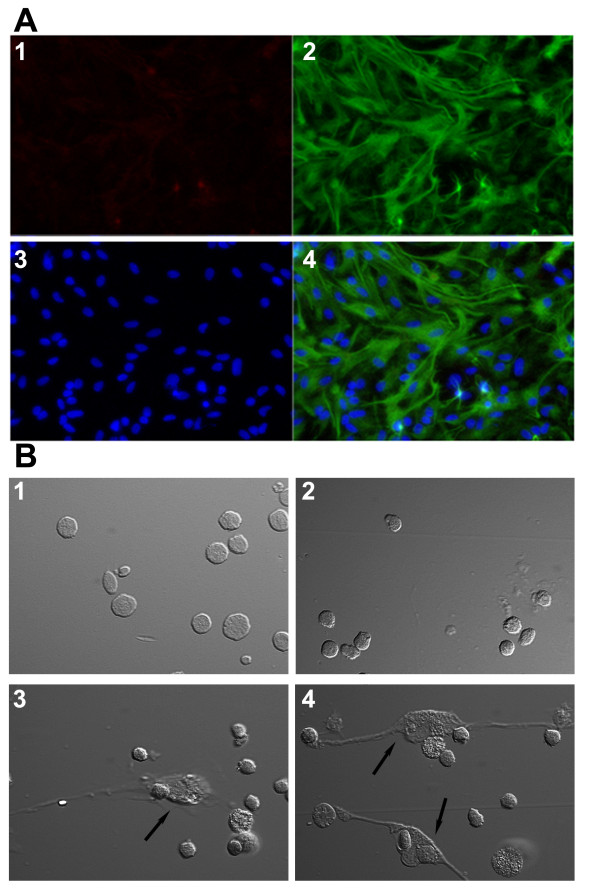
**Interactions of astrocytes and neutrophils**. ***A***. Immunocytochemical staining of F4/80 (panel 1 in red) and GFAP (panel 2 in green) in primary astrocyte culture. Cells were counterstained with DAPI (panel 3 in blue). The merged image is shown in panel 4. ***B***. Differential interference contrast images of neutrophils cultured alone or co-cultured with astrocytes for 1.5 hr. ***1***. Resting neutrophils alone; ***2***. LPS-activated neutrophils; ***3***. Resting neutrophils co-cultured with resting astrocytes; ***4***. Resting neutrophils co-cultured with poly I:C-primed astrocytes. Arrows indicate astrocytes.

We determined the purity of our primary astrocyte culture by immunocytochemistry. Primary astrocyte cultures were stained with both astrocyte and microglial markers. Immunocytochemistry demonstrated strong GFAP staining with minimal F4/80 in the primary astrocyte culture (Figure 1A). A clear change of neutrophils morphology was observed when neutrophils were co-cultured with astrocytes. Resting neutrophils maintained a round and flat appearance when cultured for 1.5 hours in plastic multiwelled plates. Upon being co-cultured with astrocytes, neutrophils appeared to be loosely attached to the plastic surface and astrocytes and a substantial increase of granular particles was found in neutrophils, which is similar to the morphological change in neutrophils as induced by LPS (Figure 1B).

### Cell-cell interaction of astrocytes and neutrophils

**Figure 2:**
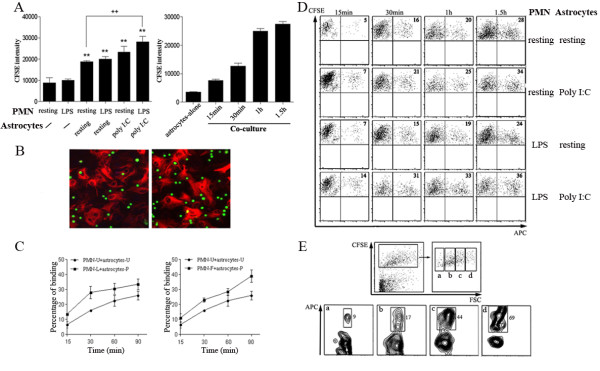
**Neutrophils bind to astrocytes *in vitro***. ***A***. FACS-sorted resting or LPS-primed neutrophils were labeled with CFSE and then were co-cultured with resting or poly I:C-primed astrocyte monolayer at a ratio of 5:1 in a 96-well plate for 1.5 hr (left) or for the indicated periods (right). CFSE intensity was analyzed (**p < 0.01 vs resting neutrophil alone; ++*p *< 0.01, LPS-primed neutrophils plus poly I:C-primed astrocytes vs resting neutrophils plus resting astrocytes. *n *= 4 - 6/bar). ***B***. After the procedure described in *A*, cells were fixed with methanol and immunoreacted for GFAP (red) followed by fluorescent microscopy. Binding of resting neutrophils (green) to resting astrocytes (left), and LPS-primed neutrophils to poly I:C-primed astrocytes (right). ***C***. Resting (PMN-U), LPS-primed neutrophils (PMN-L) or fMLP-primed neutrophils (PMN-F) were co-cultured with CFSE-labeled and resting (astrocyte-U) or Poly I:C primed astrocytes (astrocyte-P) at a ratio of 5:1. After incubation for indicated periods, neutrophil-astrocyte clusters were identified as CFSE^+^Gr1^+ ^events. Percentage of astrocytes binding to neutrophils was quantified by flow cytometry. ***D***. Representative flow cytometry graphs of ***C***. Numbers in the quadrants show the percentages of astrocytes binding to neutrophils, respectively. ***E***. Flow cytometry analysis indicates that astrocyte size is critical for binding capacity of neutrophils. CFSE^+ ^astrocytes were gated into four groups based on their forward scatter intensity: small (a), median (b), large (c) and largest (d). The numbers show the percentages of astrocytes binding to neutrophils.

We examined whether neutrophils could directly interact with astrocytes. Neutrophils were labeled with CFSE and added to cultures of confluent primary astrocytes. After 1 hour incubation, non-adherent neutrophils were washed off and CFSE intensity was determined. Significantly higher CFSE intensity was detected in all co-culture groups compared with both resting neutrophils alone and primed neutrophils alone cultures. The highest CFSE intensity was observed in co-cultures of primed astrocytes with primed neutrophils (Figure 2A left). Time course analysis showed a time-dependent increase of CFSE intensity in the co-cultures, suggesting neutrophils might bind to astrocytes through ligand-receptor engagement in a time-dependent manner (Figure 2A right). For immunocytochemistry, astrocytes were fixed and stained with Alexa Fluo594 conjugated GFAP antibody. We observed that neutrophils bind to not only astrocytes but also plastic surface, and both binding were enhanced by astrocytes activation (Figure 2B). To further determine the specific cell-cell binding, APC-conjugated-Gr1-antibody-labeled neutrophils were incubated with CFSE-labeled primary astrocytes in floating culture, and the neutrophils-astrocytes clusters were detected by FACS analysis. It demonstrated that neutrophils bind to astrocytes in a time-dependent manner (Figure 2C). Consistently, incubation of neutrophils with poly I:C-activated astrocytes significantly enhanced the direct binding of neutrophils and astrocytes (Figure 2C &2D). We found that binding ability between astrocytes and neutrophils was correlated to the size of astrocytes. The larger the astrocytes, the more neutrophils binding were observed (Figure 2E).

### Astrocytes prolong neutrophils survival dependent on cell-cell contact

**Figure 3:**
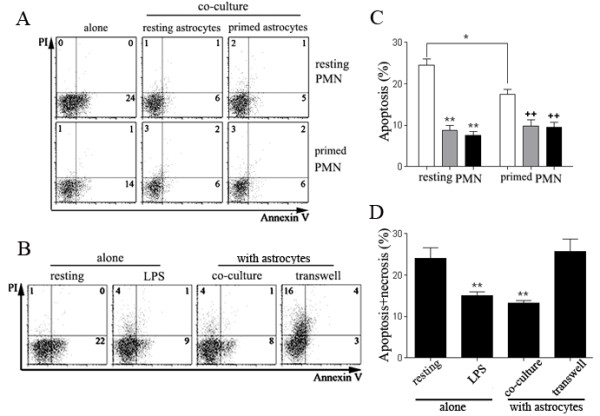
**Astrocytes prolong neutrophil survival in a cell-cell contact-dependent manner**. ***A & C***. FACS-sorted resting or LPS-primed neutrophils were co-cultured with resting or poly I:C-primed astrocytes at a ratio of 1:1 for 16 hr. After co-culture, all cells were trypsinized, washed with PBS once and the neutrophils were immunoreacted with APC-conjugated anti-mouse CD11b antibody. CD11b^+ ^Neutrophils were then stained with Annexin V and propidium iodide (PI) for detection of apoptosis and necrosis. White bar, neutrophils alone; gray bar, neutrophils co-cultured with resting astrocytes; black bar, neutrophils co-cultured with primed astrocytes (*p < 0.05, vs resting neutrophils alone; **p < 0.01, vs resting neutrophils alone; ++*p *< 0.01, vs primed neutrophils alone. *n *= 5/bar). ***B & D***. Resting neutrophils were cultured alone or cultured with astrocytes in the same (co-culture) or separate compartment (transwell) of a transwell plate for detection of apoptosis and necrosis. Neutrophils cultured in the presence of LPS were used as a positive control. Co-cultured neutrophils were collected as above and cell death was quantified with flow cytometry. (**p < 0.01, vs resting neutrophils alone).

We determined the effect of astrocytes on neutrophils survival. Neutrophils were pre-activated with LPS or fMLP for 2 hours, then, were co-cultured with primary astrocytes. After 18 hours, apoptotic neutrophils were analyzed by Annexin V/PI staining and quantified with FACS. We found that resting neutrophils terminate their life through apoptosis, not necrosis, as very few PI-positive cells were seen in resting neutrophil group. In LPS-primed neutrophils, Annexin V^+^/PI^- ^cells, which were early apoptotic cells, decreased by ~1/3 compared with resting cells. As expected, co-culture with astrocytes dramatically decreased Annexin V^+^/PI^- ^cells, indicating that astrocytes could effectively inhibit neutrophils apoptosis in an activation-independent manner. In addition, a slight increase of Annexin V^+^/PI^+ ^and Annexin V^-^/PI^+ ^neutrophils was found in co-cultures, suggesting astrocytes might either weakly accelerate the transition of neutrophils from early apoptosis to late apoptosis, or slightly promote neutrophils necrosis (Figure 3A &3C).

To investigate whether the inhibitory action of astrocyte on neutrophils apoptosis is depended on direct cell-cell binding or mediated by soluble factors, neutrophils and primary astrocytes were co-cultured at a ratio of 1:1 for 18 hour in the same or separate compartments of a transwell plate with 0.4 μm pore size polycarbonate membranes. Then, neutrophils were stained with Annexin V/PI and quantified with FACS. A similar amount of early apoptotic cells was detected in resting neutrophils. The apoptosis of neutrophils was decreased by a half upon LPS stimulation. On the other hand, a moderate increase of necrosis (PI^+^) induced by LPS stimulation was detected. Consistently, a robust inhibition of apoptosis in neutrophils was identified when neutrophils were co-culture with primary astrocytes in the same compartment. Transwell culture of neutrophils with astrocytes without direct contact demonstrated an inhibitory action on neutrophils apoptosis and a dramatic enhancement on neutrophils necrosis (Figure 3B &3D). We concluded that astrocytes could significantly enhance neutrophils survival by inhibiting both apoptosis and necrosis. The inhibitory action of astrocytes on neutrophils apoptosis was independent of cell-cell binding, while, the inhibition of necrosis was cell-cell binding-dependent. In addition, astrocytes could induce neutrophils necrosis potentially through releasing soluble factors, which could be overcome by cell-cell contact between astrocytes and neutrophils.

### Astrocytes enhance neutrophils phagocytosis

**Figure 4:**
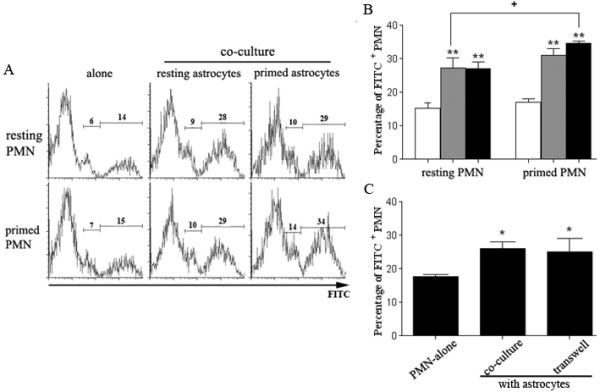
**Astrocytes enhance neutrophil phagocytosis**. ***A & B***. FACS-sorted resting or LPS-primed neutrophils were co-cultured with resting or poly I:C-primed astrocytes at a ratio of 1:1 for 6 hr. FITC-E. coli was added to the co-culture system. All cells were trypsinized and washed with PBS once. Uptake of E. coli by Gr1^+ ^neutrophils was quantified by flow cytometry analysis. Numbers indicate the percentage of FITC^+ ^neutrophils including neutrophils with E. coli adhesion to the cell surface (small middle peaks in each graph) and neutrophils with engulfed E. coli inside (right peaks in each graph). White bar, neutrophils alone; gray bar, neutrophils co-cultured with resting astrocytes; black bar, neutrophils co-cultured with primed astrocytes (**p < 0.01, vs resting neutrophils alone; +*p *< 0.05, resting neutrophils plus resting astrocytes vs primed neutrophils plus primed astrocytes. *n *= 3/bar). ***C***. Resting neutrophils were cultured alone or co-cultured with astrocytes in a transwell plate and phagocytosis of FITC-E. coli was determined by flow cytometry analysis. (*p < 0.05, vs resting neutrophils alone. *n *= 3/bar).

Phagocytosis of pathological particles as bacteria, mycoplasma, fungi and viruses is one of the main functions of neutrophils. We speculated that astrocytes might affect phagocytotic capacity of neutrophils. Co-culture with either resting or primed astrocytes, a significant increase of FITC^+ ^cells was detected, suggesting that astrocytes might enhance the phagocytotic capacity of neutrophils. In addition, the augmentary effect of astrocytes on neutrophils' phagocytosis seems independent of astrocyte activation, as no further enhancement was demonstrated in poly I:C-stimulated astrocytes compared with resting astrocytes. Co-culture of primed neutrophils with astrocytes resulted in the most FITC^+ ^cells compared with all other groups, indicating that astrocytes might promote and amplify the signals for phagocytosis in primed neutrophils (Figure 4A &4B). We concluded that resting and primed astrocytes have comparable capability to enhance phagocytotic action of resting neutrophil.

We used transwell plates to determine whether the augmentary effect of astrocyte in neutrophil's phagocytosis was mediated by direct astrocyte-PMN contact. It demonstrated that even without direct intercellular contact, the percentage of FITC^+ ^cells in transwell culture was significantly higher than that in neutrophils alone control. Furthermore, no change was found between co-culture and transwell culture of astrocytes and neutrophils, indicating that the enhancement of phagocytosis by astrocyte was mediated through astrocyte-derived soluble factors (Figure 4C).

### Astrocytes regulate neutrophils respiratory burst

**Figure 5:**
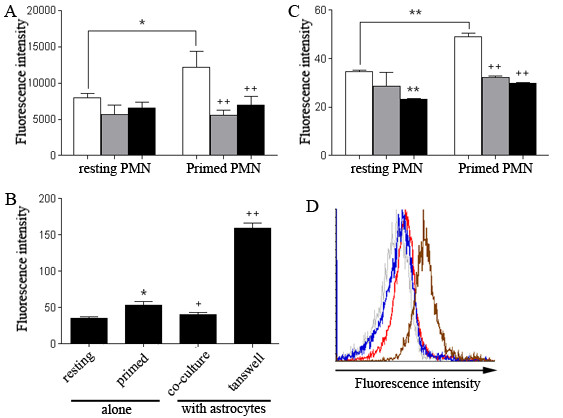
**Astrocytes reduce intracellular ROS production of neutrophils**. ***A & C***. FACS-sorted resting and LPS-primed neutrophils were stained with H2DCFDA and then were co-cultured with resting or poly I:C-primed astrocytes at a ratio of 1:1 for 6 hr. H2DCFDA intensity was then determined with a microplate reader (***A***) or flow cytometry analysis (***C***). White bar, neutrophils alone; grey bar, neutrophils co-cultured with resting astrocytes; black bar, neutrophils co-cultured with primed astrocytes (*p < 0.05, vs resting neutrophils alone; **p < 0.01, vs resting neutrophils alone; ++*p *< 0.01, vs primed neutrophils alone. *n *= 4/bar). ***B***. Intracellular ROS production by LPS-primed neutrophils was analyzed using a transwell plate and H2DCFDA intensity was quantified by flow cytometry. Resting neutrophils were used as a negative control (*p < 0.05, primed neutrophils alone vs resting neutrophils alone; +*p *< 0.05, primed neutrophils plus astrocytes vs primed neutrophils alone; ++*p *< 0.01, transwell cultured primed neutrophils vs primed neutrophils alone. *n *= 4/bar). ***D***. Representative flow cytometry graph of ROS production by neutrophils. Grey, resting neutrophils; red, LPS-primed neutrophils; blue, co-cultured primed neutrophils; brown, transwell-cultured primed neutrophils.

We subsequently detected whether astrocytes affect respiratory burst of neutrophils. Measurement of extracellular ROS production in the neutrophil-astrocyte co-culture does not precisely represent the neutrophil respiratory burst as astrocytes also express NADPH oxidase subunits. Therefore, ROS production was monitored using intracellular H2DCFDA staining and analyzed by both a plate reader and flow cytometry. In resting neutrophils, astrocytes triggered weak and insignificant decrease of basal ROS production. In LPS-primed neutrophils, both resting and primed astrocytes down-regulated intracellular H2DCFDA intensity close to the baseline, indicating their inhibitory effects on neutrophils ROS production. (Figure 5A &5C).

In transwell cultures, consistently, active astrocytes inhibited neutrophils respiratory burst through direct contact. However, a robust up-regulation of fluorescence intensity was observed when primed neutrophils and primed astrocytes were cultured in separate compartments in transwell plates, suggesting that primed astrocytes may release soluble mediators to enhance neutrophils respiratory burst (Figure 5B &5D). We speculated that neutrophils ROS production was regulated by not only surface molecules of astrocytes but also astrocyte-derived soluble factors, and the action mediated by astrocyte surface molecules could overcome the impact mediated by the soluble factors.

### Astrocytes down-regulate neutrophils degranulation

**Figure 6:**
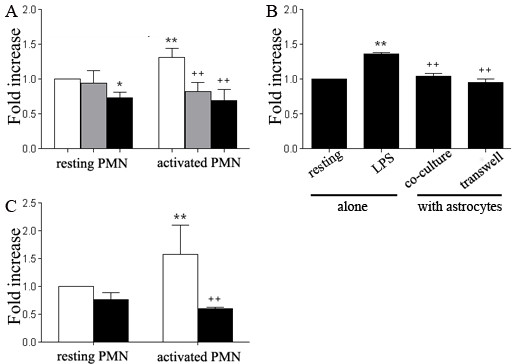
**Astrocytes inhibit MPO release from neutrophils**. ***A***. FACS-sorted resting or LPS-activated neutrophils were co-cultured with resting or poly I:C-primed astrocytes at a ratio of 1:1 for 6 hr. Semi-quantification of MPO shows fold change of MPO compared with resting neutrophils alone. White bar, neutrophils alone; grey bar, neutrophils co-cultured with resting astrocytes; black bar, neutrophils co-cultured with primed astrocytes (*p < 0.05, vs resting neutrophils alone; **p < 0.01, vs resting neutrophils alone; ++*p *< 0.01, vs activated neutrophils alone. *n *= 6/bar). ***B***. MPO release by LPS-activated neutrophils was determined in a transwell plate following the same procedure as above. Resting neutrophils were as negative control (**p < 0.01, vs resting neutrophils alone; ++*p *< 0.01, vs activated neutrophils alone. *n *= 4/bar). ***C***. Resting or LPS-activated neutrophils were incubated in the presence or absence of equal volume of astrocyte-conditioned medium followed by semi-quantification of MPO release. White bar, neutrophils alone; black bar, with astrocyte-conditioned medium. (**p < 0.01, vs resting neutrophils alone; ++*p *< 0.01, vs activated neutrophils alone. *n *= 5/bar).

Enzymes released from pre-formed granules in neutrophils, including matrix metalloproteinases, lysozymes, serine proteinases, and myeloperoxidase (MPO), play a critical role in neutrophil-mediated pathogens homicidal action as well as tissue liquefaction. We investigated the effect of astrocytes on neutrophils degranulation by analyzing released MPO within the supernatant. LPS significantly increased MPO release compared to resting neutrophils cultures. Astrocytes profoundly reduced MPO production from both resting and activated neutrophils, suggesting that astrocytes could prevent degranulation of primary granules in neutrophils (Figure 6A). Furthermore, the transwell experiment demonstrated that the inhibitory action of astrocytes on neutrophils degranulation was mediated by astrocyte-derived soluble factors independent of cell-cell contact as the attenuation of MPO releasing could be observed when astrocytes and neutrophils were cultured in separate compartments (Figure 6B). Consistently, astrocyte-conditioned media elicited similar inhibitory actions on neutrophils degranulation, as indicated by reduced MPO quantity in the supernatants (Figure 6C).

### Astrocytes regulate cytokine expression in neutrophils

**Figure 7:**
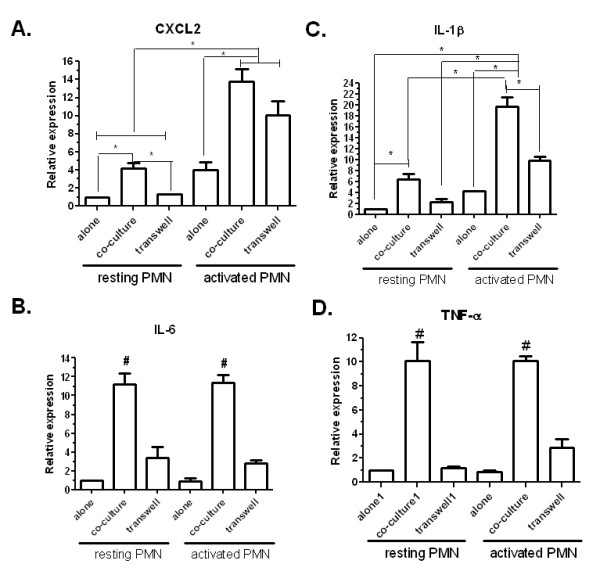
**Astrocytes regulate pro-inflammatory cytokine expression in neutrophils**. Neutrophils were isolated with magnetic Dynabeads followed by co-culture with resting astrocytes in the presence or absence of LPS for 2 hr. Neutrophils were separated from astrocytes using magnetic isolation again. Q-PCR was applied to detect mRNA levels of corresponding cytokines. (*p < 0.05 vs other indicated groups. # p < 0.05 vs all other groups).

We determined the effect of astrocytes in neutrophil-expressed pro-inflammatory cytokines including CXCL2; IL-1β; IL-6; and TNF-α, by quantitative PCR (Figure 7). Co-culture with astrocytes induced significant up-regulation of these cytokines expressed in both resting and active neutrophils. Co-culture with astrocytes further enhanced expression of CXCL2 and IL-1β in LPS primed neutrophils, suggesting a synergistic effect of astrocytes with LPS. The transwell experiment suggested that the regulatory effects of astrocytes on cytokines expression were mediated through both cell-cell contact-dependent and independent mechanisms, as the augmentary effects of astrocytes in neutrophils cytokines expression were more or less attenuated when astrocytes and neutrophils were cultured in separated compartments in a transwell system (Figure 7).

### Astrocytes induce cell signaling in neutrophils

**Figure 8:**
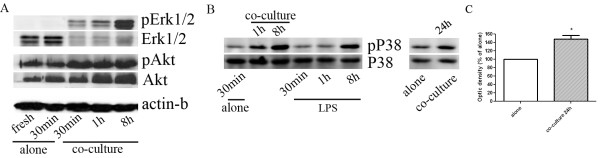
**Astrocytes induce cell signaling in neutrophils**. Neutrophils were isolated with magnetic Dynabeads. Then neutrophils were co-cultured with astrocytes for indicated periods followed by isolation with a magnetic concentrator. After confirmation of little astrocyte contamination by microscopy, neutrophil lysate was prepared and interest proteins were detected with western blot assay. ***A***. Akt/PKB and Erk1/2 activation; ***B***. left, p38 MAPK activation within 8-hr incubation. LPS-activated neutrophils are used as positive controls; right, p38 MAPK activation at 24-hr post-co-culture. ***C***. Quantitative analysis of pp38 in neutrophil lysate derived from culture alone or co-cultured with astrocytes for 24-hr. (*p < 0.05 vs alone).

To investigate the impact of astrocytes on signal transduction of neutrophils, isolated resting neutrophils were co-cultured with astrocytes and cell lysates were prepared for Western blots of Akt/PKB, Erk1/2 and p38. Co-culture with astrocytes induced up-regulation of both Akt and phosphorylated Akt levels, compared with basal levels in freshly isolated 30-min cultured neutrophils. Akt level was increased in a time-dependent manner, while, phosphorylation of Akt peaked at 30 min and maintain at a high level for at least 8 hours (Figure 8A). For Erk1/2 MAPK pathway, astrocytes increased phosphorylated Erk1/2 levels while decreased total Erk1/2 expression in neutrophils. Further, a time-dependent up-regulation of Erk1/2 phosphorylation in neutrophils induced by astrocytes were seen (Figure 8A). P38 mediates LPS-induced activation of neutrophils. Consistently, our data showed that LPS induced phosphorylation of p38 over time. Co-culture with astrocytes also increased phosphorylated p38 level in neutrophils, suggesting the involvement of p38 MAPK pathway in the regulatory action of astrocytes on neutrophils (Figure 8B left). Moreover, the augmentation of phosphorylated p38 in neutrophils by astrocytes lasted up to 24 hours (Figure 8B right and 8C). These data suggested that astrocytes might affect neutrophils biology through different signaling pathways.

## Discussion

Neuroinflammation involves participation of different cellular types from the immune system and resident CNS cells depending on the pathological condition. For acute brain damage such as stroke, traumatic brain injury, and infection et al., inflammation comprises infiltration of neutrophils and other peripheral immune cell types into the injured brain parenchyma, activation of astrocytes and microglias, and expression of proinflammatory cytokines, adhesion molecules, and other inflammatory mediators. Under these conditions, neutrophils are generally the first leukocyte subtype recruited to the brain. There they face the sophisticated CNS cellular network of which astrocytes are a critical component. Within the CNS, astrocytes outnumber neurons by approximately ten-fold and have critical roles in CNS homeostasis [20]. It is also generally accepted that astrocytes are involved in the neuroinflammatory process by secreting various cytokines such as IL-1β, IL-6 and TNF-α [21-23]. In addition, astrocytes are able to modulate functions of other cell types such as microglia and monocytes through direct cell-cell interaction [24]. Astrocytes interact with infiltrated T cells to induce development of regulator T cells so as to suppress effector T cell functions during autoimmune encephalitis [25]. Astrocytes can down regulate monocytic/microglial activation [26]. Our current study provides the first evidence that astrocytes can differentially regulate neutrophil functions, supporting the idea that astrocytes participate in neuroinflammatory responses triggered by a variety of insults [12].

We speculate that astrocytes might impact neutrophil functions through both soluble mediators and cellular contact. Cell adhesion assay confirmed direct binding of neutrophils to astrocytes. Further, more robust binding was observed between the primed astrocytes and primed neutrophils, suggesting the engagement of cell surface molecules for this interaction. In addition, our study demonstrated a significant change in neutrophil morphology induced by both resting and primed astrocytes. An alternation of cell shape is often accompanied with neutrophil mobilization and thus is considered an activation marker [27]. Consistently, microscopy revealed greater numbers of neutrophils adherent to astrocytes, suggesting that the adhesion capacity of neutrophils is upregulated by astrocytes.

Neutrophils undergo constitutive or spontaneous apoptosis as a mechanism to maintain immune system homeostasis. Importantly, neutrophil apoptosis is accompanied by a general decrease in cell function and proinflammatory capacity, which is the key to non-inflammatory removal of effect cells [10]. In the apoptosis assay, both resting and primed astrocytes were able to inhibit neutrophil apoptosis, indicating that astrocytes possess intrinsic and activation-independent ability to enhance neutrophil survival. Although the adhesion assay showed that more neutrophils bind to astrocytes when they were both primed, co-culture of primed neutrophils with primed astrocytes failed to elicit stronger inhibition of apoptosis compared to co-culture of resting neutrophils with resting astrocytes, suggesting that the molecular engagement responsible for neutrophil-astrocyte adhesion may not play an important role in the inhibitory action of astrocytes on neutrophil apoptosis. Our transwell assay demonstrated that neutrophil apoptosis was reduced, while necrosis was dramatically increased by astrocytes. We conclude that astrocytes might regulate neutrophil survival in two distinct ways: soluble factor(s) may induce neutrophil necrosis while cellular contact inhibits both necrosis and apoptosis. Thus the fate of neutrophils might depend on their distance from astrocytes. Considering the dispersed distribution of a large number of astrocytes and their numerous processes in the CNS, it is plausible that neutrophils may live longer with the aid of astrocytes. The inhibition of neutrophil necrosis might be protective for the CNS tissue as necrotic neutrophils release their cargo, including MMPs; serine proteases; elastase; and myeloperoxidase, all of which are tissue-damaging.

Apoptosis of neutrophils can be initiated by both extrinsic and intrinsic pathway stimuli. In addition, neutrophils also undergo apoptosis after phagocytosis, a process known as phagocytosis-induced cell death (PICD) [10]. Given the anti-apoptotic action of astrocytes on neutrophils, we predicted that astrocytes would inhibit phagocytotic actions of neutrophils. To our surprise, our study demonstrated that phagocytosis of neutrophils was enhanced by astrocytes through cell-cell contact-independent mechanisms. The precise mechanisms underlying this enhancing action of astrocytes on neutrophil survival and phagocytosis, and the significance of this action, are not clear. Neutrophils are phagocytic cells that are able to phagocytose and destroy infectious agents. Therefore, we speculate that the enhancing action of astrocytes on the survival and phagocytosis of neutrophils could provide potential protection against the increased risk of microbial infection due to a compromised BBB induced by stroke or traumatic brain injury. In addition, the augmentary action of astrocytes on neutrophils could limit replication of viruses or bacteria under infectious condition [28]. Neutrophils produce numerous cytokines following phagocytosis, which contribute to the inflammatory response [10]. Consistent with the augmenting action of astrocytes on neutrophil phagocytosis, our study also demonstrates that astrocytes can enhance pro-inflammatory cytokine expression in neutrophils.

Neutrophil microbiocidal processes are mediated by a combination of ROS, various hydrolytic enzymes, and antimicrobial factors [29]. It is speculated that neutrophils might utilize the same mechanisms in the CNS as in the periphery. The production of bacteriocidal products by neutrophils, including proteases, phospholipase, nitric oxide, hydrogen peroxide, and superoxide, could induce further damage to tissue that survives the initial insult. Neutrophil contact and protease production has been involved in neuron death in hippocampal primary cultures, indicating that neurotoxic agents are contained mainly in neutrophil azurophilic granules [30]. Reactive oxygen intermediates and hypohalous acid interact with an unlimited number of macromolecules, while degranulation causes the release of multiple proteases to liquefy tissue [2]. In our assay, we utilized H2DCFDA to detect intracellular ROS rather than extracellular ROS because phagocyte oxidase complex-derived ROS production by astrocytes might interfere with the quantity of neutrophil-derived extracellular ROS [31]. We found ROS production in neutrophils was reduced by astrocytes, indicating an inhibitory action of astrocytes on neutrophil respiratory bursts. This inhibition of ROS production could lessen brain damage, therefore benefitting brain recovery. Furthermore, ROS actively participates in cell signaling to influence phagocytosis, apoptosis and gene expression [32,33], indicating that astrocyte-induced ROS reduction might intertwine with signal transduction pathways to regulate neutrophil behaviors. Our results further reveal that the inhibitory effect of astrocytes on neutrophil ROS production is cell-cell contact dependent, suggesting an engagement of cell surface molecules between neutrophils and astrocytes. The inhibitory action of astrocytes on MPO release from neutrophils indicates that astrocytes may block discharge of azurophilic granules from neutrophils. Given the neurotoxic effects of neutrophils, it is speculated that astrocytes could provide protection against excessive tissue damage induced by the infiltrated neutrophils in the CNS.

Many cell signaling pathways regulate the inflammatory response and are implicated in the function of neutrophils. The mitogen activated protein kinases (MAPKs) and phosphatidylinositol 3-kinase (PI3-K) are two of the major kinases that participate in cell signaling for the growth and differentiation of neutrophils and its response to stress [34]. The serine/threonine kinase Akt is one important signal transduction pathway that mediates the delay of neutrophil apoptosis caused by inflammatory mediators [35]. Activation of p38 is required for TNF-α-induced FMLP-mediated signaling, while, ERK activation is necessary but not sufficient for neutrophil homotypic aggregation [34]. Not surprisingly, the multifaceted actions of astrocytes on neutrophil functions are associated with multiple signaling pathways. Our study demonstrates that both PI3-K and MAPK pathways in neutrophils are activated when they are co-cultured with astrocytes. However, given the different cell-cell contact-dependent and -independent actions of astrocytes on neutrophils, we predicted that astrocytes might modulate cell signaling pathways in neutrophils differently depending on the direct or indirect interaction between these two cell types.

In summary, the current study reveals sophisticated regulatory actions of astrocytes on neutrophils. Astrocytes are able to interact with neutrophils directly and indirectly, and the interaction can be enhanced by activation. Our study suggests that astrocytes can regulate neutrophil functions differently depending on direct or indirectly interaction between the two cell types. Upon direct cell-cell contact, astrocytes attenuate neutrophil apoptosis, respiratory bust, and degranulation, while, enhancing neutrophil phagocytotic capability and pro-inflammatory cytokine expression. For indirect interactions with neutrophils, astrocytes attenuate apoptosis and enhance necrosis in neutrophils, augment neutrophil phagocytosis and respiratory burst, and inhibit neutrophil degranulation. We speculate that, in vivo, proximal astrocytes regulate neutrophils through cellular contact, while distal astrocytes influence neutrophils via soluble mediators. The diversified actions of astrocytes on neutrophils could provide protection against potential microbial infection given compromised BBB integrity, and prevent excessive damage caused by neutrophil infiltration. Our results warrant further in vivo studies to determine the impact of astrocyte-neutrophil interactions in neuropathological conditions. The complex actions of astrocytes on neutrophils could provide further insight to harness the inflammatory response to promote CNS repair.

## Competing interests

The authors declare that they have no competing interests.

## Authors' contributions

LX conceived the project in discussion with AG, YW, JSW, and SY. AG provided expertise for astrocytes culture. LX, ECP, WL, AES and RL performed the neutrophil and astrocytes cultures. LX and RL performed the immunocytochemistry. LX and AES performed adhesion analysis, respiratory bust detection, phagocytosis assay, and degranulation assay. LX performed quantitative real-time PCR, western blots, and statistic analysis. LX and SY prepared the manuscript. All authors have read and approved the final version of this manuscript.
